# Mechanism by Which Heat Treatment Influences the Acoustic Vibration Characteristics of Bamboo

**DOI:** 10.3390/ma18235335

**Published:** 2025-11-26

**Authors:** Rongzhen Song, Ying Li, Shanyu Han, Lei Chen, Shumin Yang, Genlin Tian, Xing’e Liu, Fuming Chen, Zehui Jiang

**Affiliations:** 1 Institute of Biomaterials for Bamboo and Rattan Resources, International Centre for Bamboo and Rattan, Beijing 100102, China; meizhen13581661026@163.com (R.S.); chenlei@emails.bjut.edu.cn (L.C.); yangsm@icbr.ac.cn (S.Y.);; 2 Key Laboratory of National Forestry and Grassland Administration/Beijing for Bamboo & Rattan Science and Technology, Beijing 100102, China; 3 School of Materials Science and Technology, Beijing Forestry University, Beijing 100083, China; 4 Jiangsu Co-Innovation Center of Efficient Processing and Utilization of Forest Resources, College of Materials Science and Engineering, Nanjing Forestry University, Nanjing 210037, China

**Keywords:** bamboo, structural characteristics, acoustic vibration, mechanism

## Abstract

The multi-layered and multi-scale refined structure of bamboo gives bamboo musical instruments a unique tonal quality. This study employed heat treatment to enhance the acoustic vibration stability of bamboo materials. The hammering method was subsequently employed for conducting multi-point impact excitation tests on instrument-grade bamboo, and the resulting vibration response was subjected to modal analysis. Next, we investigated the acoustic vibration characteristics of bamboo, including its sound vibration efficiency, timbre, and acoustic stability, in terms of its macroscopic gradient structure, ultra-microstructure, molecular scale, key components, and pore structure. Modal analysis revealed that the first three damping ratios of Xipi were 94.55%, 7.89%, and 26.60% higher than those of Erhuang, respectively. The relative stiffness of Xipi across the first three modes was 1.22, 1.22, and 1.18 times that of Erhuang, indicating a generally higher structural rigidity. The first three natural frequencies of Xipi were approximately 1.20, 1.20, and 1.19 times higher than those of Erhuang, and its fundamental transfer function value was 1.5 times greater, suggesting a lower susceptibility to low-frequency resonance. Modal shapes showed distinct vibration behaviors between the two types: Xipi exhibited a more effective energy transmission path in the second mode and less structural distortion in the third mode, potentially indicating higher structural integrity. This research provides support for developing new technologies to select and process bamboo materials for musical instruments.

## 1. Introduction

Musical instruments are important tools and carriers for music and cultural dissemination, and the oldest in history are bamboo instruments [1,2,3,4,5,6]. Bamboo can be used as a natural musical instrument material due to its unique arrangement of fiber cells, porous structure, and high-molecular-weight components [7,8,9,10,11]. Bamboo musical instruments have important historical, cultural, and artistic value, and their most prominent features (i.e., unique sound quality and timbre) are closely related to the unique structure of bamboo itself [12,13,14]. As the world’s second-largest forest resource, bamboo is extensively distributed across Asia, Africa, Latin America, and additional areas. It has a rapid growth cycle (3–5 years to complete), high mechanical strength, wear resistance, and can be easily processed into musical instruments such as bamboo flutes [15,16,17].

The vibration performance of bamboo is an important performance indicator for its application in musical instruments. Many studies have explored the laws and factors influencing bamboo’s acoustic vibration from both macroscopic and microscopic perspectives [18,19,20,21,22]. In terms of the macroscopic gradient structure, the green part of bamboo contains materials with a higher volume fraction of cellulose, a higher Young’s modulus, faster sound velocity, and better vibrational performance [23]. From the microscopic structure of cell walls, the microfibril angle stands as the main factor that governs the vibrational performance of bamboo materials. With the reduction of the microfibril angle, the dynamic elastic modulus of bamboo increases, whereas its loss factor decreases correspondingly [24]. In terms of chemical composition changes, the vibration performance is influenced by the content, arrangement, and distribution of amorphous substances such as cellulose, which constitutes the cell wall skeleton. Additionally, it is influenced by lignin and hemicellulose, substances that constitute the matrix of the cell wall. Elevated cellulose content and a more orderly molecular chain configuration decrease the tendency for friction against other substances, ultimately contributing to the enhancement of the material’s vibration performance [25]. Furthermore, a more orderly and denser arrangement of cellulose molecular chains in the crystallization zone increases the number of internal hydrogen bonding networks. This makes it more difficult for the molecular chains to slip, resulting in less internal friction and better vibration performance. Unlike cellulose, hemicellulose and lignin exhibit an amorphous structure. Due to the presence of numerous free hydroxyl groups, hemicellulose readily absorbs ambient moisture, which in turn undermines the stability of the material’s acoustic and vibration performance. Lignin, on the other hand, is the crust material of cell walls and the main source of rigidity and viscoelasticity changes, which affect the amplitude and frequency distribution of bamboo acoustic vibrations [26]. Many studies have investigated the vibration mechanism of bamboo, mainly from the perspectives of macroscopic structure, microscopic structure, and chemical composition. However, there are few reports on how internal factors influence bamboo’s vibration performance. In current research, it has been shown that bamboo is extremely sensitive to environmental temperature and is prone to cracking due to dryness and mold growth due to moisture. This physical instability makes it difficult to maintain the pitch accuracy and timbre of musical instruments over an extended period, posing challenges to performance and preservation.

**Figure 1:**
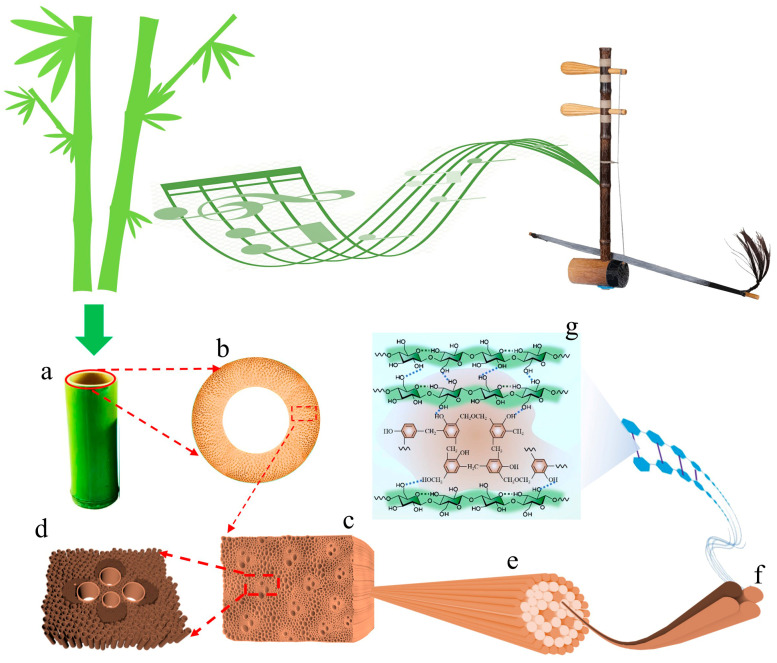
Multi-scale structure and chemical composition coordination mechanism of the acoustic vibration performance of bamboo-based musical instruments. (**a**,**b**) Gradient structure of vascular bundles from bamboo green to bamboo yellow. (**c**) Multi-scale pore structure. (**d**) Pores inside fiber cells. (**e**) Microfibril bundles. (**f**) Original fibers. (**g**) Molecular structure of cellulose.

Heat treatment is a physical modification method that is used for bamboo drying, as well as color and size stability adjustment, which benefits from its simple process, convenient control, cleanliness, and eco-friendliness. Research on heat treatment has mainly focused on modified bamboo, but there is little research on how it impacts the vibration performance of the original bamboo tube and elevates the acoustic and vibrational performance of music equipment. Therefore, with natural and pollution-free bamboo as the research subject, dynamic response tests were carried out on musical instrument-specific bamboo materials before and after heat treatment using a DASP-V11 vibration analyzer (Beijing Orient Institute of Noise & Vibration, Beijing, China). The results were used to construct an inherent structure-performance relationship between bamboo’s structure and its acoustic vibration characteristics. By characterizing changes in the chemical composition, moisture content, crystallinity, microfibril angle, FT-IR (Study on the chemical changes of samples caused by heat treatment), and porosity distribution of bamboo, the mechanism responsible for its acoustic stability improvement and vibration mode characteristics was determined (Figure 1). This work analyzes bamboo tubes used in traditional musical instruments and establishes correlations between their material characteristics and vibrational properties. The proposed mechanism provides a foundation for understanding the acoustic behavior of bamboo-based instruments. These findings could inform the future scientific selection and processing of bamboo for musical instruments, with the potential to enhance their performance and durability.

## 2. Materials and Methods

### 2.1. Materials

#### 2.1.1. Production of Bamboo Harp Tube

(1) Material selection and pretreatment

We chose bamboo with a long growth cycle and a thick yellow layer, and adjusted its inner diameter according to the tuning requirements. The inner diameter of the Xipi harp tube was 42–44 mm, and the inner diameter of the Erhuang harp tube was 45–52 mm (The musical instruments represented by Xipi and Erhuang bamboo pipes are Jinghu, mainly used in Beijing Opera accompaniment, solo performances, ethnic bands, and music education and cultural inheritance).

The fibers were softened in boiling water for 4–6 min, and wooden supports were inserted to prevent shrinkage and deformation. Then, the specimen was heated in a microwave oven for 2–3 min to thoroughly dry it.

(2) Planning and tuning

The thickness of the tube wall was adjusted with a planer, and the resonance effect was assessed by tapping the tube body. If the bamboo wall is too thick, the sound will be muffled, and if it is too thin, the sound will be scattered. The wall thickness should be controlled within the range 5.5–6.5 mm. Accurate positioning was required before drilling, so a cross-line was drawn on the cylinder body, with a hole spacing of 40 mm and 41.5 mm, respectively. This ensured that the insertion angle of the load matched the sound quality.

#### 2.1.2. Piano Stem Production

(1) Material selection and shaping

Purple bamboo from Fujian, Jiangxi and other regions was selected, with protruding bamboo nodes and dense fibers, a diameter of 18–20 mm, and a total length of five sections adjusted according to the desired tuning: Xipi harp 370–390 mm and Erhuang harp 400–430 mm.

(2) Grill straightening

After coating the bamboo pole with industrial wax, it was heated in a honeycomb coal oven to 70–80 °C and then clamped and rotated with a rounding clamp to correct the ellipticity to within 0.5 mm. The inclined hole of the stool was used to straighten it, and the shaping process was repeated 3–5 times.

(3) Shaft hole and head

Accurate positioning is required for drilling and ironing shaft holes, so a cross-line was drawn in the middle of the first section. The front and rear shaft holes were drilled with diameters of 10 mm and 12 mm, respectively. The final dimensions of the Xipi and Erhuang bamboo tubes were 113 mm and 121 mm, respectively, with a wall thickness of 7 mm and an inner diameter of 50 mm. The end conditions were smooth and uniform.

### 2.2. Experimental Methods

#### 2.2.1. Two-Dimensional Wide-Angle X-Ray Diffraction

Natural bamboo and heat-treated bamboo were ground into 60-mesh powder via a micro-grinder. After being dried at 100 ± 3 °C, their crystal structures were analyzed by 2D-WAXS (HomeLab, Rigaku, Tokyo, Japan) under optimized parameters: scanning range of 5–50° (2θ), scanning speed of 2°/min, Cu-Kα radiation (λ = 0.1541 nm) as the incident light, tube voltage of 40 kV, and tube current of 40 mA. Additionally, a Hypix-6000 photon direct-reading detector (HomeLab, Rigaku, Tokyo, Japan) (operating voltage: 40 kV, current: 40 mA) was used to acquire the wide-angle X-ray diffraction patterns of the multi-wire bundle.

The relative crystallinity was determined using a 60 mesh powder sample with a scanning angle range of 5–50°, a voltage of 40 kV, a current of 40 mA, a step rate of 0.5/step, and a step size of 0.5°. Origin software (OriginPro 2021) was used for subtracting the baseline and fitting. Five samples were tested repeatedly, and the average of the results was taken.

The crystallinity index (Crl) was calculated using the Segal formula (Equation (1)):

(1) $$Crl\;\left(\%\right)=\frac{I_{002}-I_{am}}{I_{002}}$$

where *I*_002_ and *I*_am_ are the peak intensities of the (002) crystal plane of cellulose and amorphous cellulose, respectively.

#### 2.2.2. Chemical Component Analysis

Changes in the chemical composition of the samples before and after heat treatment were determined using a Fourier-transform infrared spectrometer (Nexus 670, Thermo Electron Corporation, Waltham, MA, USA). The samples were prepared in both attenuated total reflection (ATR) and powder modes using potassium bromide tablets, and the wavenumber range of the infrared spectra was 400–4000 cm^−1^. OMNIC 8.0 software was used to perform automatic baseline correction and to smooth the obtained FTIR spectra. We tested 5 samples and selected the group with the highest data repetition rate.

#### 2.2.3. Pore Structure Characterization

High-resolution X-ray micro-CT (Nano Voxel-3000, Tianjin Sanying Precision Instrument Co., Ltd., Tianjin, China) was operated at a tube voltage of 60 kV, a current of 100 μA, and a spatial resolution of 2.4 μm. Each bamboo sample (4.0 mm (T) × 4.0 mm (L) × 1.0 mm (R)) underwent a 90 min scan. Avizo 3D software (Amira 2019.1) was then used to perform 3D reconstruction, thereby obtaining the internal pore structure and porosity of the samples prior to and following heat treatment. Five test specimens were used, and the results were averaged.

#### 2.2.4. Scanning Electron Microscopy

A field emission environmental scanning electron microscope (FE-ESEM, FEI Corporation, Hillsboro, OR, USA)—featuring a maximum resolution of 2 nm—was employed for characterizing the morphological properties of the samples. The experiment was conducted at a test voltage of 20 kV, using samples with dimensions of 5 mm × 4 mm × 1 mm (length × width × height). Before testing, the sample surface was cleaned with ethanol, subjected to vacuum gold spraying, and the sample chamber was kept in a dry condition throughout the experiment.

#### 2.2.5. Acoustic Vibration Characteristic Testing

**Figure 2:**
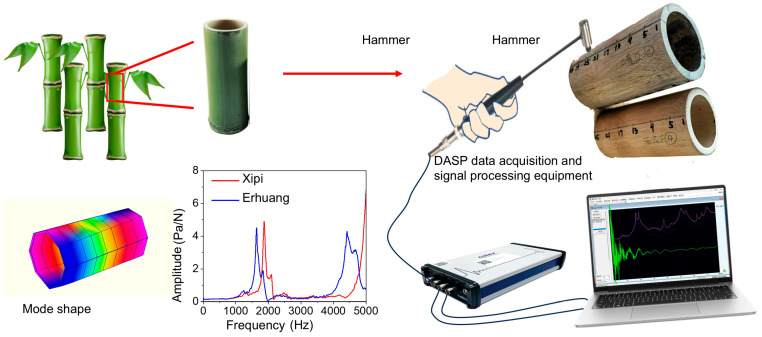
Schematic diagram of the measuring point arrangement and device for vibration modal testing of bamboo for musical instruments.

As shown in Figure 2, According to the “Measurement of Sound Insulation in Buildings and Building Components” (GB/T 19889.3-2005) [27], a DASP instrument (V11, China Eastern Institute of Noise & Vibration, Shanghai, China) was used to characterize the vibration behavior of the material under a dynamic load. Multiple-point impact excitations were applied to Xipi and Erhuang using the hammer method to test their vibration response characteristics and complete the modal analysis. The measurement system was composed of acquisition and analysis software, signal acquisition instruments, and sensing devices. The software module included PolyLSCF basic modal analysis software and PolyIIR modal analysis software (DASP V11). For signal acquisition, the INV3062T device was adopted, and the sensing components included an ICP force hammer (INV9310) and two ICP sound pressure sensors (INV9206) with a measurement range of 50 g. The sample was divided into four equal parts within the excitation area circumference, for a total of 36 measuring points. For the damping ratios and frequencies of Erhuang and Xipi, five specimens were tested separately for each type, and the experimental results were averaged. To obtain information with a high signal-to-noise ratio, sampling points 15 and 36, which were not close to the nodes, were selected as sampling points. Sound pressure sensors were installed at each sampling point. To mitigate the effect of constraints on measurement results, the boundary condition was configured as free vibration, with a support structure consisting of four soft rubber ropes arranged at the four ends of the sample. A force hammer shaker was used to sequentially strike from point 1 to point 36, exciting each position five times to obtain response signals for each point. The excitation signal obtained from the accelerometer was transmitted to the dynamic analyzer for analysis and calculation. The frequency response function curve of the sample was acquired through the transfer function approach. Following this, modal identification analysis was conducted to obtain the first three order natural frequencies, damping ratios, and modal configurations. Based on the identified natural frequencies, the equivalent stiffness of the specimen was estimated using Equations (2) and (3).

(2) $$K-\omega^{2}M=0$$

(3) $$\left|\frac{K_{1}}{K_{2}}\right|=\left|\frac{\omega_{1}^{2}M_{1}}{\omega_{2}^{2}M_{2}}\right|$$

where K is the first-order relative dynamic stiffness; ω is the first-order natural frequency; M is the relative mass.

## 3. Results and Discussion

### 3.1. Influence of the Gradient Structure of Bamboo Vascular Bundles for Musical Instruments on the Acoustic Vibration Performance

**Figure 3:**
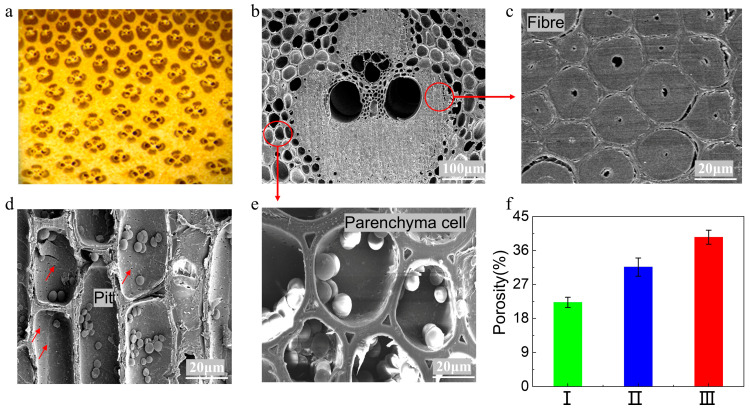
Gradient structure of vascular bundles in bamboo: (**a**) Gradient structure of vascular bundle thin-walled cells in the transverse section of bamboo; (**b**) SEM image of bamboo vascular bundle; (**c**) SEM image of fibroblast cross-section; (**d**) SEM image of fiber cell diameter section; (**e**) SEM image of thin-walled cell cross-section; (**f**) Porosity distribution of the bamboo cross-section.

From a macroscopic perspective, bamboo is a natural composite composed of rigid vascular bundle fibers and thin-walled cells that are combined in a series-parallel manner. In the radial direction of bamboo, the number and size of vascular bundles exhibit a gradient distribution from the green side to the yellow side: the number decreases from “many to few”, while the size increases from “small to large” (Figure 3a). The green side is characterized by dense vascular bundle fibers, which are tightly arranged between cell wall layers and adjacent cells, with extremely small cell lumens and cell wall pores (Figure 3d). In contrast, the yellow side has a higher proportion of thin-walled cells with large cavities, accompanied by loose and porous interlayer walls, as well as abundant intercellular spaces and cell wall pores (Figure 3e).

The gradient structure of vascular bundles contributes to bamboo’s acoustic vibration characteristics through two primary aspects. First, vascular bundle fibers, especially solid fiber sheaths (Figure 3c), provide dynamic stiffness that supports efficient elastic wave propagation. Second, thin-walled cells, with their loose and porous structure (Figure 3b) and abundant cell wall matrix materials, introduce damping effects that regulate energy dissipation. Collectively, the complementary interaction between these two cell types creates a two-phase composite system that fundamentally shapes bamboo’s acoustic vibration behavior; the stiff vascular bundles facilitate elastic wave transmission, while the porous thin-walled tissues modulate vibrational energy.

At the microscale, the porosity across the bamboo wall exhibited a gradient increase along the radial direction, from 22.25 ± 1.36% in the outer layer to 31.62 ± 2.42% in the middle and 39.54 ± 1.86% in the inner layer (Figure 3f). This porosity gradient suggests a corresponding gradient distribution in stiffness and damping properties. The stiffness gradient likely results in a spatial variation of sound velocity and resonance frequency, which could promote multiple local resonances across a broad frequency range. Furthermore, the structural combination of low-damping bamboo green, characterized by higher radiation efficiency, and high-damping bamboo yellow, which helps suppress resonant peaks, creates a complementary system in the Jinghu bamboo. This integrated structure achieves an effective balance between wideband sound radiation and peak suppression, forming the physical basis that may contribute to the instrument’s distinctive timbre and acoustic projection.

At the microscopic level, pores larger than 50 nm in bamboo may function as sound transmission channels and resonance cavities, potentially contributing to its acoustic characteristics. Meanwhile, pores smaller than 50 nm, located within lignin and at the interfaces between hemicellulose and the cellulose skeleton in the cell wall, are expected to provide damping and energy dissipation effects on the vibration and energy transmission of cellulose microfibrils. The synergistic interaction between these microstructural features and the macroscopic organizational characteristics of bamboo likely plays a fundamental role in shaping its unique acoustic vibration properties.

### 3.2. Modal Vibration Performance of Bamboo for Musical Instruments

The vibrational characteristics of bamboo musical instruments are highly dependent on their geometric dimensions and material properties. According to Euler-Bernoulli beam theory, the fundamental frequency is inversely proportional to the square of the length. Literature reports that for Moso bamboo tubes approximately 1 m in length and 80–100 mm in diameter, the fundamental frequency typically falls within the range of 80–200 Hz [25]. In contrast, the Jinghu stems (Xipi and Erhuang) investigated in this study are greatly shorter. Based on this scaling law, their whole modal frequencies are expected to be considerably higher than the typical values for 1 m tubes. Furthermore, the complex vibrations of instrument tubes under practical boundary conditions, which may involve coupling between whole bending, torsion, and local drum-like modes, result in a modal sequence that deviates from that of a simply supported homogeneous beam model [28].

**Figure 4:**
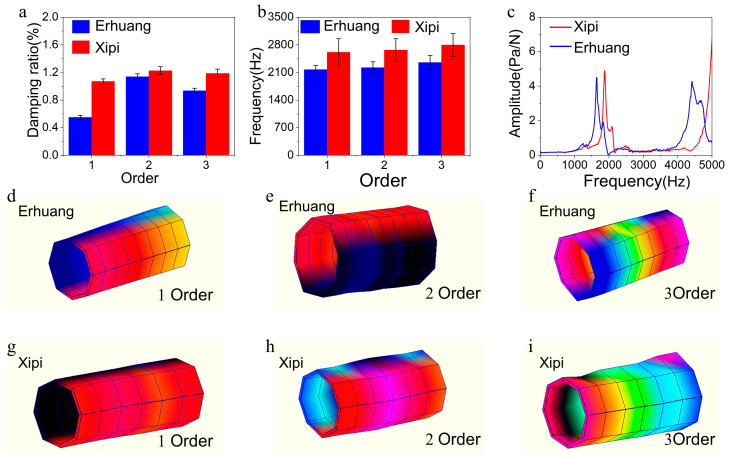
Damping, stiffness, and frequency response of bamboo in musical instruments. (**a**) Damping ratio of bamboo-based musical instruments. (**b**) The frequency of materials used for bamboo-based musical instruments. (**c**) The relative stiffness of bamboo-based musical instrument materials. (**d**–**f**) Third-order modal shape diagram of Erhuang. (**g**–**i**) Xipi’s third-order modal shape diagram.

The damping ability of materials is influenced by their stiffness and damping characteristics, where increasing either the stiffness or the damping improves the energy loss of vibrations. To analyze the vibration performance of Xipi and Erhuang, the first three vibration modes of Xipi and Erhuang were analyzed using the transfer function method. Figure 4a shows that the first three damping ratios of Xipi were 1.07 ± 0.04% (1st order), 1.23 ± 0.06% (2nd order), and 1.19 ± 0.06% (3rd order), which were 94.55%, 7.89%, and 26.60% higher than those of Erhuang (0.55 ± 0.03%, 1.14 ± 0.04%, and 0.94 ± 0.04%, respectively). Figure 4b shows the relative stiffness and the relative stiffness-to-mass ratio of the first three orders of Xipi obtained from the physical and mechanical properties of Erhuang. The relative stiffness of Xipi’s first three orders was 1.22, 1.22, and 1.18 times that of Erhuang, respectively. This indicates that the improvement in the material’s vibration damping capacity was dominated by the increase in stiffness and offset the decreased damping. Compared with Erhuang, Xipi’s increased stiffness also improved its vibration damping capacity. The high stiffness and the measured high-frequency modes of bamboo tube identify it as an efficient high-frequency resonator. The gradient structure from the outer to inner layer plays a critical role: the dense bamboo green provides the stiffness required for high-frequency vibration, while the porous bamboo yellow, with its higher damping, helps smooth the frequency response and suppress harsh resonant peaks, which may lead to a pure timbre.

Figure 4c depicts the frequency response functions corresponding to the first three orders of both Xipi and Erhuang, wherein Xipi exhibited a higher fundamental frequency and lower vibration response. Under the different size factors in this study, increasing the stiffness also increased the fundamental frequency of Xipi. The first three fundamental frequencies were 2611 ± 343 Hz, 2670 ± 285 Hz, and 2797 ± 292 Hz, which were 1.20, 1.20, and 1.19 times greater than those of Erhuang (2173 ± 101 Hz, 2221 ± 147 Hz, and 2355 ± 170 Hz), respectively. This is consistent with the damping ratio law of the first three sections. An increase in fundamental frequency may enable materials to avoid resonance caused by low-frequency vibrations caused by human activities. The frequency response function stands as an inherent characteristic of materials, and when a material is subjected to random excitation, the response it produces is a linear superposition of all order modes excited by external forces. Lower-order modes have a greater effective mass and are more easily excited. Therefore, engineering applications are more affected by fundamental frequency modes with a larger effective mass. The transfer function value at the fundamental frequency can be used to evaluate the damping characteristics of a material (Figure 4c). The fundamental transfer function value of Xipi was 6.80 Pa/N, while that of Erhuang was 4.5 Pa/N, indicating that Erhuang should vibrate less when excited. Xipi had a higher fundamental frequency, making it less prone to resonance, and its response in the low-frequency range was lower, indicating superior low-frequency vibration suppression.

The modal shape reflects the vibration response form of a specimen at various resonant frequencies and is determined by the mass distribution and stiffness distribution. Figure 4d–f and Figure 4g–i show the first three modal shapes of Erhuang and Xipi, respectively. The vibration modes of the two were upper and lower drum-shaped. The first three vibration modes of the sample were a four-sided torsional drum (1st order, Figure 4d,g), a middle bending drum (2nd order, Figure 4e,h), and a coupled bending-torsion drum (3rd order, Figure 4f,i). The first-order torsional drum vibration mode showed that the amplitude of the Erhuang drum was larger and consumed more vibrational energy. For the 2nd-order bending drum vibration mode, the intense red resonance band of Xipi is mainly distributed at both ends of the composite board, while that of Erhuang is concentrated in the middle of the board (Figure 4e). This indicates that the vibration energy absorbed by Xipi can be transmitted to and released from both ends. In the 3rd-order bending-torsional coupled drum vibration mode, two yellow-red resonance bands are notably transmitted from both ends toward the middle. Additionally, the torsional amplitude of Erhuang is smaller than that of Xipi.

In general, the identified high-frequency modes are critical to the Jinghu’s vibrational behavior. The excitation of these modes likely results in strong high-frequency radiation, contributing to the instrument’s bright timbre and powerful acoustic penetration.

### 3.3. Acoustic Vibration Mechanism of Bamboo in Musical Instruments

**Figure 5:**
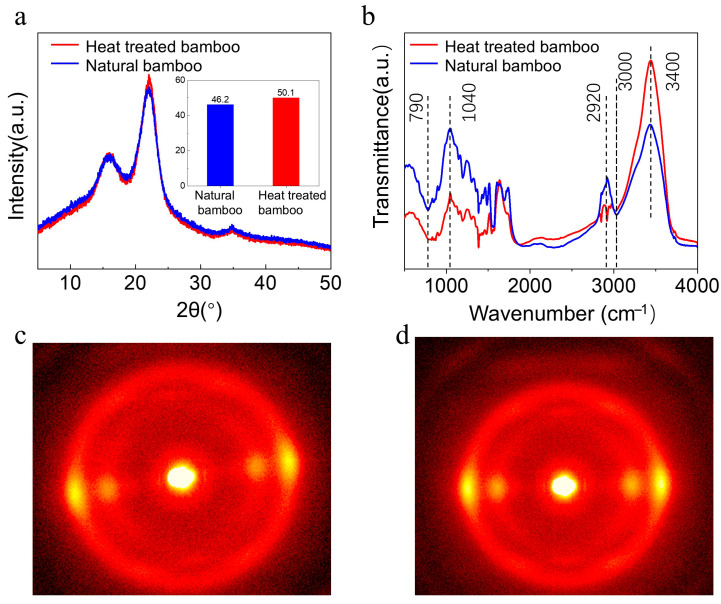
Physical and chemical properties of bamboo used for musical instruments before and after heat treatment. (**a**) XRD patterns and crystallinity values before and after heat treatment. (**b**) FTIR spectra before and after heat treatment. 2D-WAXS diffraction pattern (**c**) before and (**d**) after heat treatment.

Figure 5 shows the XRD patterns, crystallinity, microfibril angle, and chemical composition differences of Jinghu bamboo before and after heat treatment. As the skeletal material that constitutes the cell wall of bamboo, cellulose determines the stiffness and strength of bamboo via the arrangement and stacking of its molecular chains. The XRD patterns (Figure 5a) show that both natural bamboo and heat-treated bamboo exhibited typical natural cellulose Iβ crystal patterns, with cellulose chains alternating between disordered amorphous regions and highly ordered crystalline regions. The relative crystallinity (Crl) describes the proportion of crystalline regions in an overall cellulose sample, with a higher crystallinity indicating a higher stiffness. The crystallinity of the original bamboo was 46.2%, and that of the heat-treated bamboo was 50.1% (Figure 5a). The increase in crystallinity is not obvious, mainly because the main target of heat treatment is the amorphous zone in bamboo, especially hemicellulose, and the cellulose in the crystalline zone is very stable under conventional heat treatment conditions, making it difficult to undergo chemical reactions that greatly increase crystallinity. During heat treatment, cellulose and hemicellulose in bamboo underwent thermal decomposition. As the temperature increased, hemicellulose was gradually degraded, and its content decreased. The crystallinity of cellulose first increased and then decreased with the temperature. The increased crystallinity improved the mechanical properties of bamboo, but the crystallinity decreased when cellulose underwent thermal degradation at high temperatures, which weakened the mechanical properties of bamboo. Heat treatment may have also changed the microstructure of bamboo’s cell walls. The crystallinity and intermolecular hydrogen bonding of cellulose increased, which may have enhanced the rigidity of the cell wall. The thermal degradation of hemicellulose in the cell wall also affected its integrity and decreased its strength after heat treatment.

Figure 5c,d present the 2D-WAXS patterns of natural bamboo and heat-treated bamboo used for Jinghu. Natural bamboo exhibits stronger diffraction peaks in the equatorial direction, indicating that the arrangement of cellulose crystalline nanostructures is enhanced during the drying process. Cellulose microfibrils are highly oriented within the secondary cell walls of bamboo fiber cells, and the alternating thickness of microfibril layers forms the structural basis of bamboo fiber cells. The S2 layer microfibrils bear most of the axial load, and their orientation is closely associated with the mechanical properties of bamboo [29].

The FTIR spectra in Figure 5b were obtained before and after heat treatment, in which the peak near 3400 cm^−1^ represents the stretching vibration of C=O bonds in hemicellulose [28]. The intensity of this absorption peak gradually decreased after heat treatment. The stretching vibration peak of C-O bonds in cellulose and hemicellulose was observed at 3000 cm^−1^ [30,31], and the absorption peak intensity after heat treatment was slightly higher than that before heat treatment. The vibration intensity of C-O-C groups in cellulose and hemicellulose observed near 2920 cm^−1^ also slightly decreased, and it can be observed that the peak at this location is relatively stable, indicating a slight decrease in the cellulose content before and after heat treatment [32]. The peaks near 1040 cm^−1^ and 790 cm^−1^ represent the aromatic skeletal vibrations and vibration of β-glycosidic bonds in cellulose of lignin, and the absorption peak width after heat treatment was higher than that before heat treatment [33,34]. The changes in the FTIR spectra of bamboo before and after heat treatment mainly reflected the degradation of hemicellulose, changes in lignin content, and changes in the intensity of hydroxyl and carbonyl absorption peaks. These changes indicate a redistribution of the chemical composition and structural changes within bamboo.

**Figure 6:**
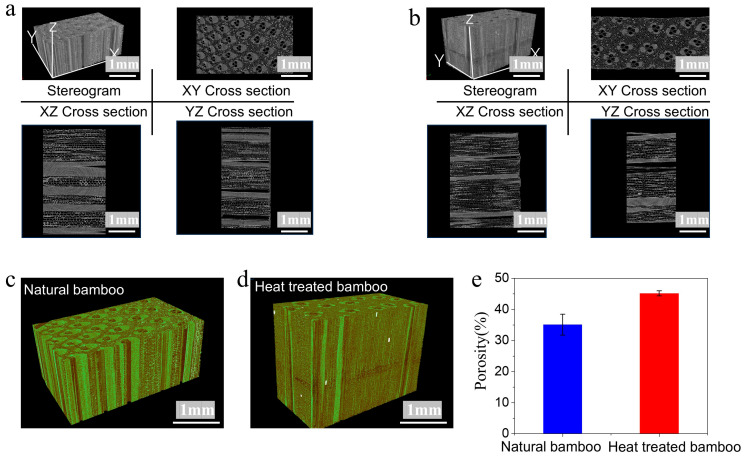
Structural changes of bamboo before and after heat treatment. (**a**,**b**) Three-dimensional images of bamboo before and after heat treatment. (**c**,**d**) Three-dimensional pore distribution of bamboo before and after heat treatment. (**e**) Porosity of bamboo before and after heat treatment.

The 3D reconstruction of the sample shows changes in the internal pores of the material. Micro-CT was performed on bamboo before and after heat treatment to characterize the internal pore structure of both samples. The images in Figure 6d show that the number of pores increased after heat treatment (Figure 6c), leading to a porosity of 45.17 ± 0.82%, which was an increase of 28.58% compared with the sample before heat treatment (35.13 ± 3.35%) (Figure 6e), and this difference was statistically significant (*p* < 0.05). The mechanism responsible for the increase in the porosity of bamboo after heat treatment involved changes in the internal structure of bamboo, the thermal decomposition of chemical components, and changes in the physical morphology. In terms of internal structural changes, bamboo is a natural non-uniform two-phase composite material composed of bamboo fibers and a matrix. Heat treatment broke connections between bamboo fibers and the matrix, causing the fibers to separate from the matrix and form a large number of pores. The moisture inside the bamboo evaporated, causing volume shrinkage and cracking. As the temperature increased, these cracks continued to propagate, which increased the porosity. Lignin and hemicellulose in bamboo underwent thermal decomposition at high temperatures. After lignin underwent thermal decomposition, some low-molecular-weight compounds were formed, which left pores inside bamboo after volatilization. The thermal decomposition of hemicellulose also loosened the internal structure of bamboo, further increasing the porosity. Cellulose underwent thermal degradation at high temperatures, which produced small molecules and gases. The escape of these gases formed pores inside bamboo, which increased its porosity. In terms of physical changes during heat treatment, a large amount of water inside bamboo evaporated, which formed pores inside bamboo, thereby increasing the porosity. Bamboo underwent repeated thermal expansion and shrinkage during cooling, which caused its internal structure to loosen and increased its porosity. The pyrolysis of hemicellulose produced loose cell walls, while the evaporation of water increased the volume of the cell lumen and intercellular spaces. The pyrolysis of lignin and cellulose further destroyed the cell wall structure, and the formation of microcracks in the cell wall caused by dry stress was the main reason for the higher porosity of heat-treated bamboo.

## 4. Conclusions

This study systematically investigated the vibrational characteristics and structural evolution of bamboo for musical instruments (Xipi and Erhuang) before and after heat treatment. The key findings are summarized as follows:

The vibrational modal analysis revealed distinct dynamic behaviors between Xipi and Erhuang. Xipi exhibited significantly higher relative dynamic stiffness and fundamental frequencies across the first three orders compared to Erhuang. This enhanced stiffness, a primary factor for the elevated natural frequencies, coupled with its specific modal shapes—particularly a more effective energy transmission path observed in the second-order mode—suggests that Xipi possesses superior resistance to low-frequency resonance and different vibrational energy dissipation characteristics. These quantified differences in dynamic performance provide a material-level explanation for their distinct operational behaviors in musical applications.

Heat treatment induced significant microstructural and chemical modifications. An increase in cellulose crystallinity and intermolecular hydrogen bonding was identified, contributing to enhanced cell wall rigidity. Concurrently, a substantial increase in porosity (28.58%) was observed, primarily attributable to the evaporation of moisture, thermal decomposition of hemicellulose, and the formation of microcracks due to drying stresses. The synergistic effect of these changes—increased rigidity alongside increased porosity—fundamentally alters the viscoelastic properties of the bamboo material. When water evaporated during the heat treatment of bamboo, pores formed inside and increased the porosity. Bamboo underwent thermal expansion and cooling shrinkage during heat treatment, and this repeated expansion and shrinkage loosened the internal structure of the bamboo, thus increasing its porosity (from 35.13% to 45.17%). While these structural changes are closely related to the observed vibrational properties, their direct impact on acoustic performance—such as timbre, sound projection, or musical quality—was not experimentally verified in this study.

The results indicated that bamboo’s acoustic vibration characteristics were collectively influenced by its oriented gradient structure, the two-phase composite structure of fiber-thin-walled tissue, the multi-scale pore structure, and the viscoelastic properties of hemicellulose and lignin in the rigid cellulose macromolecular chain. These findings provide a robust scientific foundation for the selective processing and application of bamboo in musical instruments and pave the way for future research aimed at directly correlating these material properties with specific acoustic qualities.

## Data Availability

The original contributions presented in this study are included in the article. Further inquiries can be directed to the corresponding authors.
